# A Bioluminescent Imaging Mouse Model for Seasonal Influenza Virus Infection Based on a Pseudovirus System

**DOI:** 10.3390/v17050686

**Published:** 2025-05-09

**Authors:** Yifei Wang, Mengyi Zhang, Yimeng An, Lanshu Li, Hao Wu, Ziqi Cheng, Ling Pan, Chaoying Yang, Weijin Huang, Yansheng Geng, Chenyan Zhao

**Affiliations:** 1 Division of HIV/AIDS and Sexually Transmitted Virus Vaccines, Institute for Biological Product Control, National Institutes for Food and Drug Control (NIFDC), WHO Collaborating Center for Standardization and Evaluation of Biologicals, Beijing 102629, China; 2 Key Laboratory of Public Health Safety of Hebei Province, School of Public Health, Hebei University, Baoding 071000, China

**Keywords:** seasonal influenza virus, bioluminescent imaging, animal model, vaccine evaluation, pseudovirus

## Abstract

Influenza (flu) is a highly prevalent respiratory illness caused by influenza viruses, representing a significant global health burden due to its substantial morbidity and mortality rate. Vaccination remains the most effective strategy for influenza prevention, and well-characterized animal models of influenza infection serve as essential tools for evaluating vaccine protective efficacy. However, animal models utilizing live influenza virus strains pose significant biosafety concerns, and many such strains are not readily available for research. To address these challenges, we established a novel visual mouse infection model using an HIV-based vector system. This model employs influenza pseudoviruses carrying a luciferase reporter gene, enabling real-time monitoring of viral load and in vivo tracking of viral distribution during infection. Using this infection model, we assessed the in vivo protective efficacy of an influenza vaccine and cross-validated the pseudovirus-based evaluation results against a live virus-infected mouse model. Our study thus establishes a safer and more convenient platform for evaluating influenza vaccine efficacy, including the assessment of broad-spectrum neutralization capacity.

## 1. Introduction

Influenza is an acute respiratory illness caused by influenza viruses, primarily transmitted via respiratory droplets, direct human contact, and fomite transmission. This disease exhibits significant epidemic potential and is frequently associated with seasonal outbreaks and global pandemics [1,2], with seasonal influenza causing between 290,000 and 650,000 deaths annually from respiratory illnesses [3]. In 2024, surveillance data from the U.S. Centers for Disease Control and Prevention (CDC) indicate that most reported human avian influenza cases resulted from direct exposure to sick or deceased poultry. Notably, four recent cases involved zoonotic transmission of influenza A (H5N1) viruses between dairy cows, marking a novel transmission route [4]. Influenza viruses are enveloped and segmented into negative-sense RNA viruses with genomes consisting of eight RNA segments (influenza A and B viruses) or seven segments (influenza C and D viruses) [5,6]. These segments encode the following major proteins: hemagglutinin (HA), neuraminidase (NA), non-structural proteins (NS1 and NS2), matrix proteins (M1 and M2), nucleoprotein (NP), polymerase acidic protein (PA), and polymerase basic proteins (PB1 and PB2) [7,8]. Among these, HA proteins represent the most abundant surface glycoproteins, forming trimeric spikes that recognize and bind to host cell sialic acid receptors (α2,6- or α2,3-linked) [9,10]. These proteins mediate both viral attachment and subsequent membrane fusion, thereby facilitating viral entry into host cells [11]. As the primary target of host immune recognition, HA effectively elicits potent neutralizing antibody responses, with induced antibody titers exceeding those of NA [12,13]. While the globular head domain exhibits substantial antigenic variation, the stem domain demonstrates remarkable evolutionary conservation (>85% sequence similarity) while preserving its essential membrane fusion function [14,15]. These characteristic high-surface exposure, strong immunogenicity, and the presence of conserved epitopes establish HA as an optimal target for rational vaccine design [16,17,18].

In the research on evaluating the effectiveness and protective efficacy of new influenza vaccines, the conventional evaluation system mainly includes two modules: in vivo evaluation (such as animal model experiments) and in vitro evaluation (serological testing) [19]. Serological testing technology uses standardized methods such as hemagglutination inhibition assays (HI), single radiation hemolysis test (SRH), virus neutralization test (VN), pseudovirus-based neutralization assay (PBNA), neuraminidase activity test, and cell-mediated immune response test, which can quantitatively analyze influenza-specific antibodies [20,21,22,23]. In the European Union, all novel influenza vaccine candidates must demonstrate compliance with stringent regulatory standards established by the European Medicines Agency (EMA). This includes successful validation through standardized serological assays, specifically the SRH and HI tests, as essential prerequisites for market authorization. In vivo models demonstrating superior capacity to recapitulate natural influenza virus infection encompass ferrets, mice, guinea pigs, cotton rats, hamsters, swine, avian, and non-human primates (NHPs) [24,25,26,27]. Among these models, mice represent the most extensively utilized system for influenza research. To establish productive infection, viral strains typically require multiple rounds of lung-adapted passage to achieve efficient replication in murine systems [28,29].

Bioluminescent imaging (BLI) technologies have revolutionized the study of infectious diseases, particularly for seasonal influenza viruses. These models allow for non-invasive longitudinal observations, providing insights into viral dynamics without the need for euthanizing animals at multiple time points. In this study, a mouse model employing a bioluminescent reporter virus has shown great promise, enabling real-time tracking of influenza infections [30,31]. Czako et al. engineered an infectious and pathogenic bioluminescent reporter virus (H1N1pdm09-NLuc) that enables immunological evaluation of both human monoclonal antibodies (hmAbs) and HA-specific antibodies through real-time monitoring of viral infection dynamics [32]. Kim et al. engineered an avian influenza virus carrying a C-terminal NanoLuc reporter fused to PB2 protein (PB2-C-NanoLuc), which enables real-time viral tracking while maintaining wild-type virulence in murine models [33]. However, these animal models universally pose inherent biosafety risks including potential aerosol transmission.

Currently, pseudovirus-based in vivo animal models are gaining increasing research attention due to their enhanced biosafety and greater accessibility to mutant viral strains. Highly pathogenic pseudovirus-based in vivo imaging mouse models have been developed for evaluating immune sera and antibodies such as SARS-COVID-2, MERS, HPV, Nipah, and Ebola virus, which shows that the use of pseudoviruses has broad application prospects [34,35,36]. Pseudoviruses are chimeric viral particles that structurally and functionally mimic live viruses while lacking their pathogenicity. These replication-deficient systems can be safely studied under biosafety level 2 (BSL-2) conditions and can be engineered to incorporate reporter genes, enabling both quantitative and qualitative analysis of viral entry and infection dynamics. Following administration, pseudoviruses enable real-time dynamic monitoring of viral load and tissue distribution patterns in vivo through bioluminescent live imaging techniques. In this study, we developed a novel visual mouse infection model using a safe lentiviral-based influenza pseudovirus system (pHIV/HA/Fluc). By integrating pseudovirus technology with bioluminescent in vivo imaging, we established for the first time a seasonal influenza pseudovirus infection model. This system enabled evaluation of in vivo vaccine efficacy across different influenza seasons. Furthermore, we created a stable live virus infection mouse model to validate our pseudovirus-based assessments. Together, these approaches provide a safer and more efficient platform for evaluating influenza vaccine efficacy and assessing broad-spectrum neutralizing antibody responses.

## 2. Materials and Methods

### 2.1. Cells, Plasmids, Vaccines, Strains, and Animals

293T (ATCC, CRL-3216), MDCK cells (ATCC^®^NBL-2), Huh7 (JCRB0403), and the plasmids used were deposited in the AIDS laboratory of the National Institutes for Food and Drug Control (NIFDC, Beijing, China), The 2022–2023 quadrivalent influenza split-virion vaccine and live virus strains (A/Victoria/2570/2019 [H1N1] and A/Victoria/4897/2022 [H1N1]) were generously provided by Hualan Biological Engineering, Inc. (Henan, China).

All animal procedures were conducted in compliance with Assessment and Accreditation of Laboratory Animal Care International (AAALAC) guidelines and were approved by the Institutional Animal Care and Use Committee of the NIFDC (Beijing, China). Approval No. 2023(B)035). Specific pathogen-free (SPF) Hartley guinea pigs, along with BALB/c, C57BL/6, KM, and NIH mice, were sourced from the NIFDC Institute of Laboratory Animal Resources.

### 2.2. Influenza Pseudovirus Packaging and Titration

The HA sequence of influenza virus was downloaded from the official website of Global Initiative on Sharing All Influenza Data (GISAID). The optimized full-length codon sequence HA was cloned into the pcDNA3.1 vector to obtain the expression plasmid pcDNA3.1-HA. The pcDNA3.1-HA and backbone plasmid pSG3Δenv.cmvFluc were co-transfected into 293T cells at a ratio of 2:1 using the transfection reagent Lipofectamine3000 (Invitrogen, Carlsbad, CA, USA). Fresh medium containing 1% FBS was added to the cells. After 6 h of transfection, the old culture medium was discarded and replaced with fresh culture medium containing 1% FBS. At the same time, 7 mU/mL of neuraminidase (Aladdin, Shanghai, China) was added to induce the release of pseudoviruses from the cell surface. After 48 h, the supernatant culture medium was collected into a centrifuge tube and centrifuged at 4000 rpm for 10 min. The supernatant was treated with 25 μg/mL TPCK-trypsin (1 mg/mL; Sigma-Aldrich, St. Louis, MO, USA) and incubated at 37 °C for 30 min to facilitate enzymatic digestion. Subsequently, bacterial contaminants and particulate matter were removed by filtration through a 0.45 μm mixed cellulose ester membrane (Millipore, Burlington, MA, USA). Processed aliquots were cryopreserved at −80 °C for long-term storage.

Firstly, the influenza pseudovirus to be tested was diluted three times in a row, and then 100 μL of the diluted pseudovirus was inoculated into a 96-well plate with MDCK cells (80~90% confluence), and an uninfected cell control was set up. The mixture was incubated in a 37 °C, 5% CO_2_ incubator for 48 h. Finally, 100 μL per well of Bright-Glo luciferase reagent (Promega, Madison, WI, USA) was added, and the relative light unit (RLU) was measured using PerkinElmer multimode microplate reader. The virus titer was defined as the highest dilution (TCID_50_/mL) that produced a positive signal in 50% of the target cells. The Reed–Muench method was used to calculate TCID_50_. All experiments required technical replicates (n ≥ 3) and independent verification.

### 2.3. Pseudovirus-Based Neutralization Assay

The immune serum was initially diluted to an appropriate concentration and subsequently serially diluted three-fold. These diluted serum samples were combined with pseudovirus and incubated at 37 °C for 1 h. The serum–virus mixtures were then transferred to MDCK cells seeded in 96-well plates and cultured for 48 h at 37 °C in a humidified 5% CO_2_ atmosphere. Neutralization titers were determined using the Reed–Muench method and are reported as the 50% inhibitory dilution (ID_50_) values.

### 2.4. Influenza Vaccine Immunization in Mice

BALB/c mice in the vaccinated group received two intramuscular injections (15 μg/100 μL total dose of quadrivalent influenza recombinant protein vaccine, containing 3 μg HA per strain) administered at 2-week intervals. Control group mice were administered with an equal volume (100 μL) of PBS via the same route.

### 2.5. Influenza Pseudovirus and Live Virus Challenge

In the pseudovirus challenge model, immunized mice received an intraperitoneal injection of 1 mL influenza pseudovirus (pHIV-HA-Fluc) 28 days post-immunization. Bioluminescence imaging was performed at predetermined time points to quantify viral load. Vaccine protective efficacy was assessed by comparing the mean luminescence intensity between immunized and naive control groups, with the reduction in signal intensity serving as a quantitative measure of vaccine-induced protection.

In the live virus challenge model, mice were intranasally inoculated with 50 μL of 10 LD_50_ influenza virus on day 28 post-immunization. Daily monitoring included the following: (1) survival rate, (2) body weight changes, and (3) clinical symptom scoring (0~5 scale) evaluating fur ruffling, hunched posture, and mobility impairment. All scoring was performed by a single investigator to ensure consistency. On day 5 post-challenge, lung tissues were collected for immunofluorescence analysis using HA-specific antibody staining to assess viral load and tissue pathology.

### 2.6. Bioluminescence Imaging

Bioluminescence imaging (BLI) was conducted using an IVIS Lumina Series III system (PerkinElmer, Waltham, MA, USA). Mice received intraperitoneal injection of D-luciferin substrate (50 mg/kg, Xenogen–Caliper Corp, Alameda, CA, USA, Cat# 122799) and were anesthetized with isoflurane 8 min post-injection. Imaging was performed 2 min after anesthesia induction using a 60 s exposure time. Quantitative analysis of regions of interest (ROIs) was performed using Living Image software (Caliper Life Sciences, Hopkinton, MA, USA), with bioluminescent signals expressed as photon flux (photons/s/cm^2^/sr) and visualized as pseudo-color images ranging from red (maximum intensity) to blue (minimum intensity).

### 2.7. Passive Immunological Evaluation

Guinea pigs received intramuscular electroporation of 100 μL pcDNA3.1-HA plasmid (A/Guangdong-Maonan/SWL1536/2019) on days 0, 14, and 28. Post-immunization sera were collected and screened for HA-specific antibodies. Sera-positive animals were stratified into concentration-gradient groups and incubated with influenza pseudovirus (37 °C, 1 h). Subsequently, 1 mL of the serum–pseudovirus mixture was administered via tail vein injection to naive mice. Viral distribution was assessed by in vivo imaging on day 5 post-challenge.

### 2.8. Statistical Analysis of Data

All statistical analyses were performed using GraphPad Prism 10.0 (GraphPad Software, San Diego, CA, USA). Data are presented as mean ± standard deviation (SD). Group comparisons were analyzed using either Student’s t-test or one-way ANOVA with appropriate post-hoc tests for nonparametric data. Statistical significance was defined as *p* < 0.05, with the following asterisk notation: * *p* < 0.05, ** *p* < 0.01, *** *p* < 0.001, and **** *p* < 0.0001.

## 3. Results

### 3.1. Construction and Optimization of Mouse Models for Bioluminescence Imaging

**Figure 1:**
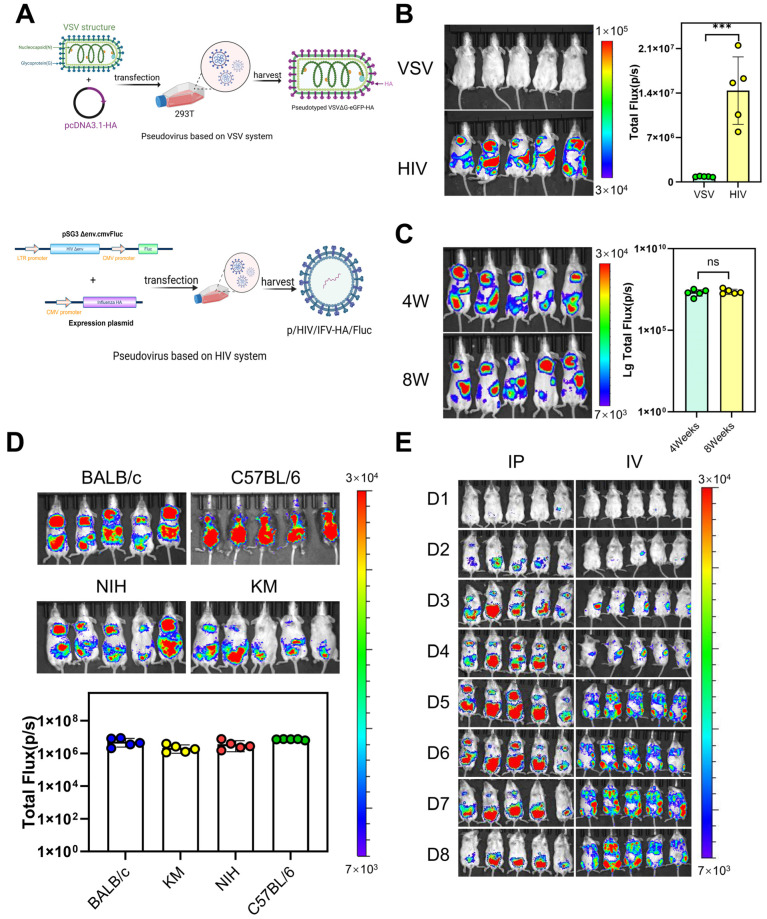
(**A**) Schematic representation of reporter plasmid constructs (pHIV-HA-Fluc and pVSVΔG-HA-Fluc) and pseudovirus packaging strategy. (**B**) Comparative susceptibility of mouse models to H1N1 influenza pseudoviruses packaged using HIV versus VSV systems. (**C**) Age-dependent infectivity comparison between 4-week- and 8-week-old mice. (**D**) Strain-specific susceptibility profiles across BALB/c, KM, NIH, and C57BL/6 mouse strains. (**E**) Route-of-infection efficiency comparison between intraperitoneal (IP) and intravenous (IV) administration. Statistical significance was defined as *p* < 0.05, with the following asterisk notation: *** *p* < 0.001, ns (not significant): *p* ≥ 0.05.

To establish a bioluminescent imaging model of seasonal influenza pseudovirus infection in mice, we firstly tested influenza pseudoviruses using the lentiviral vector packaging system (p/HIV/HA/Fluc) and vesicular stomatitis virus packaging system (p/VSVΔG/HA/Fluc) constructed earlier in our laboratory (Figure 1A). The results showed that p/HIV/HA/Fluc pseudovirus had a significant infection effect on mice (Figure 1B). Next, we tested the sensitivity of mice of different ages (4 weeks, 8 weeks) and 4 different mouse strains (BALB/c, C57BL/6, KM, NIH) to the H1N1 influenza pseudovirus. The results showed that there was no significant difference in the infectivity of mice at different ages (Figure 1C). All four strains of mice showed strong signals (Figure 1D). Since the black hair of C57BL/6 mice would block part of the light emission during imaging, BALB/c mice were more strongly infected and more uniform, so BALB/c mice were selected as the animal model. We compared the intraperitoneal injection and tail vein injection routes. The results showed that the intraperitoneal injection route had obvious specific recruitment of aggregates in the mouse lungs, so intraperitoneal injection was selected as the subsequent injection method (Figure 1E). The luminescence phenomenon of the intraperitoneal injection route was continuously monitored, and it was found that the fluorescence in the mice began to decrease on the 6th day, so the 5th day was selected as the detection time.

**Figure 2:**
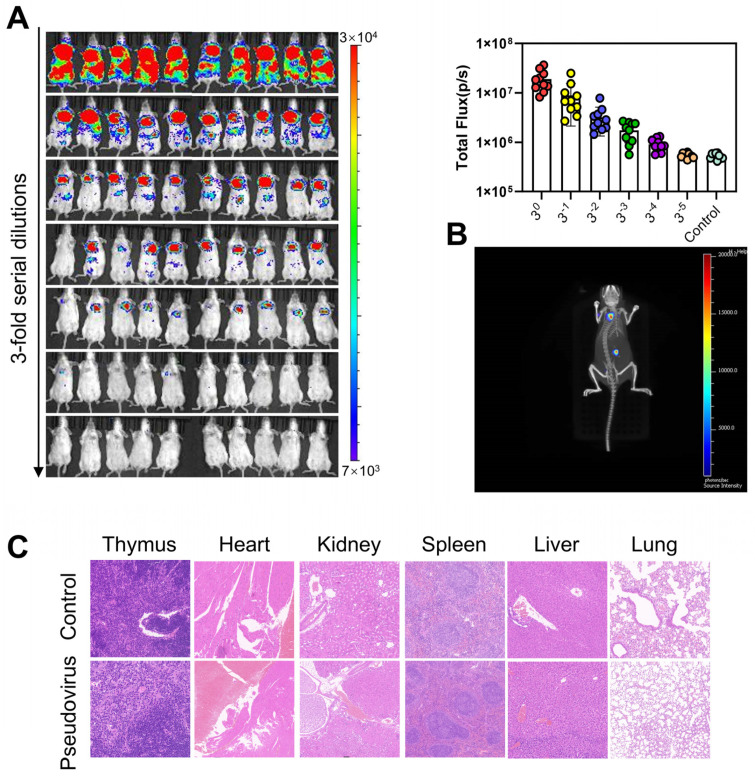
(**A**) Determination of 50% mouse infectious dose (ID_50_) using serial 10-fold dilutions (3^0^~3⁵ TCID_50_/mL) of H1N1 pseudovirus (1 mL volume, *n* = 10/group). PBS-injected controls (*n* = 10) served as negative controls. Bioluminescence signals were quantified at day 5 post-infection. (**B**) Three-dimensional biodistribution mapping of pseudovirus infection at day 5. (**C**) Histopathological evaluation by H&E staining comparing lung and spleen sections from pseudovirus-infected versus PBS-control mice, demonstrating preserved tissue architecture.

In addition, 1.24 × 10^6^ TCID_50_ of influenza pseudovirus was serially diluted from 3^0^ to 3^5^ series, and the half-infectious dose of influenza pseudovirus to mice was determined to be 1.2 × 10^5^ TCID_50_ (Figure 2A). Three-dimensional reconstruction of whole-body bioluminescence imaging revealed successful viral infection without apparent pathology (Video S1), as confirmed by hematoxylin and eosin (H&E) staining of lung and spleen sections (Figure 2B,C). Histopathological examination showed preserved tissue architecture with no evidence of virus-induced damage.

### 3.2. Evaluation of Vaccine Protection Using a Live Virus Infection Animal Model

**Figure 3:**
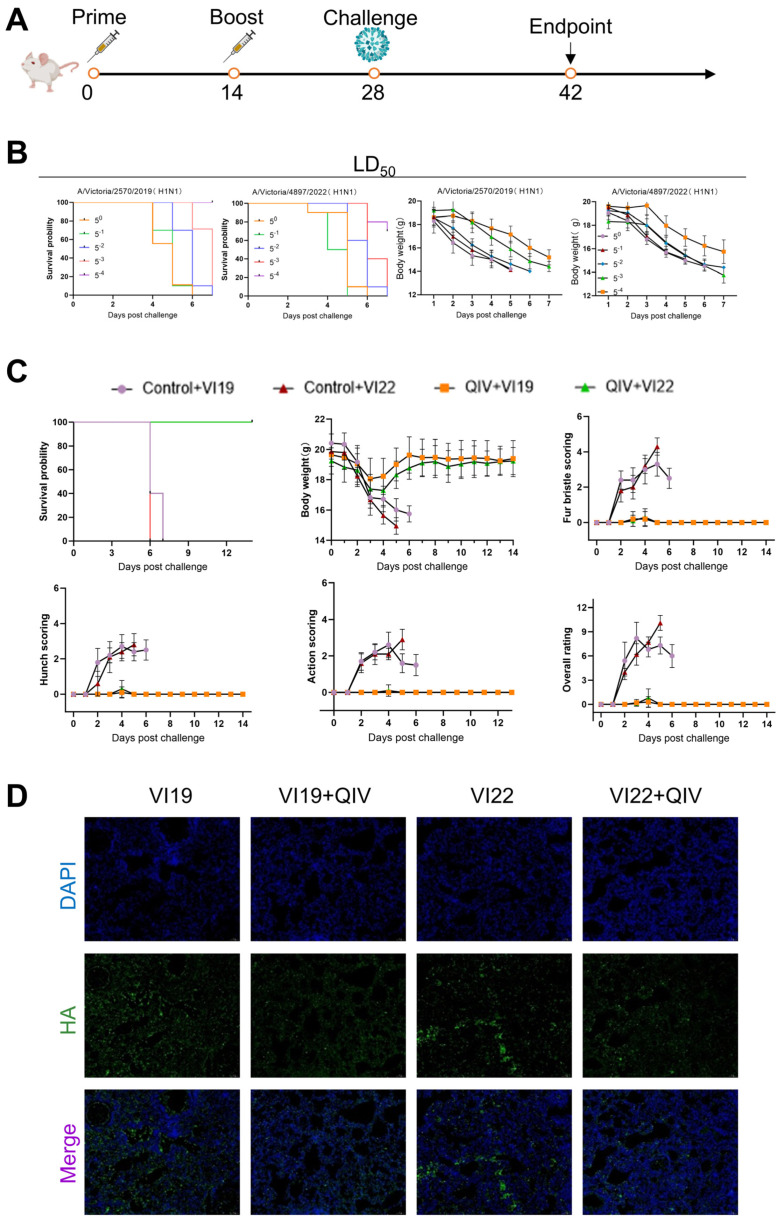
Assessment of vaccine efficacy in live influenza challenge models. (**A**) Immunization schedule. (**B**) Viral lethality determination (LD_50_) for strains VI19 and VI22. (**C**) Protective efficacy evaluation post-challenge. (**D**) HA-specific immunofluorescence (green) in lung sections 5 days post-infection.

Firstly, the protective effect of the 2022–2023 quadrivalent influenza vaccine was tested in a live virus-infected mouse model. We used WHO-recommended northern hemisphere egg-based candidate vaccine strains H1N1 (A/Victoria/2570/2019) “VI19” (2022–2023) and H1N1 (A/Victoria/4897/2022) “VI22” (2023–2024) to infect animal (Figure 3B). The mice were divided into immunized and non-immunized groups. Mice were immunized with quadrivalent influenza vaccines according to the immunization schedule (Figure 3A). The mice were infected intranasally with VI19 and VI22 at a dose of 10 LD_50_. The survival, weight changes, and clinical sign scores (hair frying, back bending, limited activity) of the mice were continuously monitored for 14 days. According to the requirements of the Association for AAALAC, a 25% weight loss is considered death. The experimental results showed (Figure 3C) that compared with the immunized group, the non-immunized group mice showed obvious symptoms from the third day after infection, including death, significant weight loss, back bending, hair erection, trunk curling and limited activity. Notably, all mice in the immunized group survived, and there was no significant change in weight and clinical symptoms. Immunofluorescence examination of lung sections of mice after attack showed that the vaccine protected mice from viral infection (Figure 3D). Furthermore, the 2022–2023 quadrivalent influenza vaccine had a 100% lethal protection rate.

### 3.3. Evaluation of Vaccine Protection Using a Pseudoviral Infection Animal Model

**Figure 4:**
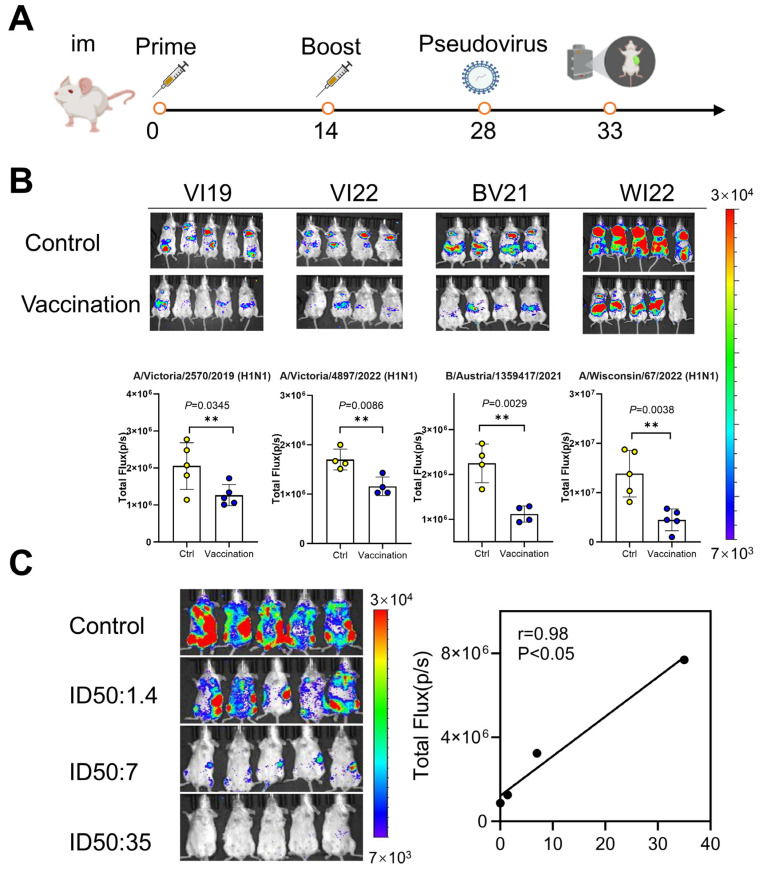
Assessment of vaccine efficacy using influenza pseudovirus models. (**A**) Immunization and challenge schematic. (**B**) Neutralization efficacy of 2022–2023 quadrivalent vaccine against egg-propagated strains (VI19, BV21,VI22) and cell-cultured strains (WI22). (**C**) Passive immunization protection analysis. ** means *p* < 0.05.

Next, we evaluated and compared the protective efficacy of the 2022–2023 influenza vaccines in our constructed pseudovirus infection animal model (Table A1). According to the immunization procedure and virus challenge procedure, our vaccine has a protective effect against VI19, VI22, A/Wisconsin/67/2022 (H1N1) “WI22” (2023~2024), and B/Austria/Australia/1359417/2021 (Victoria) “BV21” (2022~2023) (Figure 4A). According to the change of photon amount in the vaccine group and unimmunized group of mice, it was shown that the 2022–2023 influenza vaccine had 38.4% protection against the current year’s strain VI19, 50% protection against BV21, 32% protection against the next year’s strain VI22, and 65% protection against WI22. The protective effect of the 2022–2023 vaccine against VI19 of the current year and VI22 of the following year was consistent with the results of the live virus infection model (Figure 4B).

Additionally, we constructed the HA-expressing plasmid for A/Guangdong-Maonan/SWL1536 “GD19” and generated immune sera from guinea pigs immunized with pcDNA3.1-A/GD19/2019-HA, achieving a neutralization titer (ID_50_) of 3500. These high-titer sera were subsequently used for passive immunization studies. For passive immunization, serum aliquots (10, 2, and 0.4 μL) were incubated with 1 mL pseudovirus (37 °C, 1 h) prior to tail vein injection in BALB/c mice. Antibody-mediated protection was quantified by luminescence intensity at day 5 post-infection. Results demonstrated significant inhibition of pseudovirus infectivity (*p* < 0.05) when pre-incubated with immune serum, showing dose-dependent neutralization. Luminescence values decreased proportionally with increasing antibody concentrations (10 μL > 2 μL > 0.4 μL), revealing a strong positive correlation between serum antibody levels and protection efficacy (r = 0.98) (Figure 4C). This model validates a reliable platform for quantifying influenza antibody-mediated protection.

## 4. Discussion

Current animal models present distinct advantages and limitations for studying influenza pathogenesis and vaccine efficacy [37]. Murine models represent the predominant system for influenza infection studies, allowing quantitative assessment of vaccine-mediated protection through survival analysis and clinical scoring following challenges with live influenza virus [38]. However, the use of live influenza viruses presents biosafety concerns due to potential aerosol transmission risks. Furthermore, the limited availability of pandemic strains and the requirement for mouse adaptation through serial lung passages significantly constrain research progress in both influenza virology and vaccine/drug development [39].

In this study, we successfully constructed the recombinant plasmid pcDNA3.1-HA by inserting the HA gene into the pcDNA3.1 expression vector. Through co-transfection with the pSG3Δenv.cmvFluc plasmid, we successfully generated high-titer influenza pseudovirus p/HIV/HA/Fluc. Based on this approach, we successfully established an in vivo bioluminescent imaging animal model for real-time monitoring of viral infection dynamics. Compared with the influenza pseudovirus packaged in the vesicular stomatitis virus p/VSVΔG/HA/Fluc system, the influenza pseudovirus packaged based on the HIV skeleton plasmid pSG3.Δenv.cmvFluc system was significantly and stably imaged in mice. The superior infectivity of HIV-based pseudovirus in mice stems from three key advantages. VSV-dependent expression systems exhibit more rapid transcriptional silencing due to promoter methylation [40,41,42]. Subsequently, through the screening of animal age, strain, infection route, detection time, and infection dose, intraperitoneal injection showed significant luminescence aggregation in the lungs of mice, and influenza virus infection also triggers inflammation in the upper and lower respiratory tracts [43]. Therefore, our final optimized protocol employed intraperitoneal injection in BALB/c mice, with in vivo imaging conducted at day 5 post-infection. The model demonstrated consistent pseudovirus infectivity across different mouse ages, confirming its stability. Figure 2A demonstrates a dose-dependent increase in photon flux correlating with escalating pseudovirus inoculum, showing significant linear correlation. The proportional signal enhancement confirms quantitative monitoring capability. This result was explained in these studies [44]. Three-dimensional imaging revealed primary infection sites in lungs and spleen, a tropism pattern identical to live influenza virus infection [45,46]. Overall, although mouse signs did not show pathogenic infection, influenza pseudoviruses can mimic the infectious properties of live viruses in mouse models, providing an alternative approach to the protective relevance of adherent and incoming vaccines.

Influenza live virus infection models are often used for preclinical evaluation of vaccine protective efficacy. Here, we used both constructed pseudovirus models and live virus models to evaluate influenza vaccine efficacy. Using the pseudovirus infection animal model to evaluate the influenza vaccine, the influenza vaccine from 2022 to 2023 had 38.4% and 50% protective efficacy against the current year’s egg-based strains VI19 and BV21, respectively, and 32% and 65% protection against the egg-based strains VI22 and the cell-based WI22. These findings aligned with results from live virus challenge studies. Notably, the pseudovirus model demonstrated superior accuracy over conventional live virus models in quantifying vaccine-induced protection, while eliminating biosafety concerns associated with pathogenic strains.

Following immunization with pcDNA3.1-HA plasmid, guinea pigs generated high-titer polyclonal antisera. Subsequent neutralization assays demonstrated potent activity against pseudoviruses, with an ID_50_ titer of 1:3,500. The serum was used to conduct passive immune protection experiments on mice. Results demonstrated that polyclonal antibodies conferred significant in vivo protection in mice (*p* < 0.05), with a strong positive correlation between serum antibody levels and protective efficacy (r = 0.98). Bioluminescence intensity exhibited dose-dependent increases corresponding to viral titer escalation. This system provides a more efficient platform for evaluating both monoclonal and polyclonal antibody responses [47,48].

BLI enables real-time viral tracking while significantly reducing the number of animals required for experimental studies. Various reporter genes, such as Nanoluc and Fluc, have been utilized in this approach [49,50,51,52,53]. For instance, Lin et al. engineered PR8-Fluc (H1N1) and X31-Fluc (H3N2) reporter viruses to longitudinally monitor infection dynamics in live animals [54]. Similarly, Pan et al. employed reverse genetics to integrate a bioluminescent reporter into replication-competent IAV, facilitating sensitive real-time analysis of viral transmission and pathogenesis [55]. The difference is that our model employs a pseudovirus system, offering enhanced safety and convenience. It also enables rapid construction of diverse subtypes to address pandemic strains and adaptive mutations.

Our innovative model addresses three key limitations of conventional approaches: aerosol transmission risks, prolonged timelines for live virus studies, and limited availability of mutant strains. Furthermore, it enables longitudinal, real-time tracking of infection dynamics within individual animals, serving as a powerful platform for both antiviral drug development and investigation of immune evasion mechanisms. Although the influenza pseudovirus animal infection model has performed well in the evaluation of vaccine protective efficacy, it also has certain limitations. For example, whether the pseudovirus model can fully reflect all immune responses in natural influenza virus infection is still a question that needs further verification. At the same time, this model can be integrated with cutting-edge immunological approaches including single-cell RNA sequencing and high-throughput antibody profiling to systematically investigate host–pathogen interactions [56]. Such multimodal analysis will provide unprecedented insights into immune response dynamics following pseudovirus challenge, enabling comprehensive elucidation of vaccine protection mechanisms.

## Data Availability

The data are contained within the article and Supplementary Materials.

## Supplementary Materials

The following supporting information can be downloaded at: https://www.mdpi.com/article/10.3390/v17050686/s1, Video S1: 3D imaging of influenza pseudovirus tissue distribution in mice.

## Appendix A

**Table A1:** Strain information.

Name	Isolate ID	Year	Egg/Cell	Abbreviation
A/Victoria/2570/2019 (H1N1)	EPI_ISL_417210	2022~2023	egg	VI19
B/Austria/1359417/2021	EPI_ISL_1519459	2022~2023	egg	BV21
A/Victoria/4897/2022 (H1N1)	EPI_ISL_16714268	2023~2024	egg	VI22
A/Wisconsin/67/2022 (H1N1)	(EPI_ISL_15928563	2023~2024	cell	WI22
A/Guangdong-Maonan/SWL1536/2019	/	2020~2021	egg	GD19
